# Molecular analysis of canine circovirus in dogs from animal shelters in Belém, Pará, northern Brazil: first detection at the amazon region

**DOI:** 10.29374/2527-2179.bjvm000723

**Published:** 2023-10-03

**Authors:** Bruna Trindade Moreira Cardoso, Danielle Rodrigues de Deus, Edivaldo Costa Sousa, Kenny da Costa Pinheiro, Jonaia Novaes da Costa, Marcelino Antonio Costa Maués, Márcia Janete de Fátima Mesquita, Dielle Monteiro Teixeira, Jones Anderson Monteiro Siqueira, Hugo Reis Resque, Yvone Benchimol Gabbay, Luciana Damascena da Silva

**Affiliations:** 1 Biomedical Scientist, Programa de Pós-graduação em Biologia Parasitária da Amazônia (PPGBPA), Centro de Ciências Biológicas e da Saúde (CCBS), Universidade do Estado do Pará, Belém, PA, Brazil.; 2 Biologist, MSc., Programa de Pós-graduação em Virologia, Instituto Evandro Chagas, Ananindeua, PA, Brazil.; 3 Biomedical Scientist, DSc., Seção de Parasitologia, Instituto Evandro Chagas, Ananindeua, PA, Brazil.; 4 Biomedical Scientist, DSc., Seção de Virologia, Instituto Evandro Chagas, Ananindeua, PA, Brazil.; 5 Veterinarian, DSc., Centro de Controle de Zoonoses do Pará, Belém, PA, Brazil.; 6 Veterinarian, DSc., Universidade Rural da Amazônia, Belém, PA, Brazil.

**Keywords:** circovirus, diarrhea, dogs, molecular characterization, circovirus, diarreia, cães, caracterização molecular

## Abstract

The canine circovirus (CanCV) is a single-stranded DNA virus that has become an important emerging virus associated with gastroenteritis in dogs worldwide. In the present study, the CanCV was detected by PCR in 15% (22/147) of dogs from animal shelters in Belém, between 2019 and 2020. We observed an association between the CanCV infection and the presence of diarrhea in animals younger than one year of age (p > 0.01). The Brazilian strains were grouped in Chinese genotypes, with 99.54 to 100% nucleotilde homology. The GMRF Bayesian Skyride used the molecular clock model, which was the best suited technique to plot the dataset. The most recent common ancestor (TMRCA) was estimated in 2017, with the evolution rate of 1.6 x 10^-3^ s/s/y. The viral family diversity was also investigated, with emphasis on the families of the enteric pathogenic viruses Parvoviridae, Picornaviridae and Astroviridae, which were detected in the CanCV positive pooled samples. This study highlights the importance of the CanCV as an emergent virus that causes diarrhea in Brazilian dogs. The results found herein contribute to the understanding of the role of CanCV in enteric diseases and in the evolutionary molecular characterization of the circulating genotypes. Furthermore, we increased the understanding of the fecal virome in dogs with diarrhea, providing data for the monitoring and prevention viral gastroenteric diseases in domestic animals.

## Resumo

O circovírus canino (CanCV) é um vírus de DNA de fita simples que se tornou um importante vírus emergente associado à gastroenterite em cães em todo o mundo. No presente estudo, o CanCV foi detectado por PCR em 15% (22/147) dos cães de abrigos de animais em Belém, entre 2019 e 2020. Observamos uma associação entre a infecção pelo CanCV e a presença de diarreia em animais menores de um ano de idade (p > 0,01). As linhagens brasileiras foram agrupadas em genótipos chineses, com 99,54 a 100% de homologia nucleotídica. O GMRF Bayesian Skyride foi o modelo de relógio molecular utilizado, sendo o método mais adequado para o conjunto de dados. O ancestral comum mais recente (TMRCA) foi estimado em 2017, com taxa de evolução de 1,6 x 10-3 s/s/ano. A diversidade de famílias virais também foi investigada, com destaque para as famílias dos vírus patogênicos entéricos Parvoviridae, Picornaviridae e Astroviridae, que foram detectadas nos pools de amostras positivas para CanCV. Este estudo destaca a importância do CanCV como um vírus emergente que causa diarreia em cães brasileiros. Os resultados aqui encontrados contribuem para a compreensão do papel do CanCV nas doenças entéricas e na caracterização molecular evolutiva dos genótipos circulantes. Além disso, aumentamos a compreensão do viroma fecal em cães com diarreia, fornecendo dados para o monitoramento e prevenção de doenças gastroentéricas virais em animais domésticos.

## Introduction

CanCV is a non-enveloped, circular and single-stranded DNA (ssDNA) virus belonging to the *Circovirus* genus, of the Circoviridae family. Its genome is comprised of approximately 2,063 nucleotides with two open reading frames (ORFs) in opposite directions, encoding the viral replicase (rep) and capsid protein, plus two non-coding regions (Zhai et al., 2014; Kapoor et al., 2012; Kotsias et al., 2019).

The CanCV was detected in dogs in the United States in 2012 (Kapoor et al., 2012), and its circulation has already been reported in different regions of the world, such as Italy (Decaro et al., 2014), Germany (Anderson et al., 2017), Thailand (Piewbang et al., 2018), South America (Weber et al., 2018; Kotsias et al., 2019; Giraldo-Ramirez et al., 2020), China (Niu et al., 2020) and Vietnam (Tuong et al., 2021). These studies demonstrate the worldwide distribution of CanCV in dogs, and evidence shows that this virus also circulates in wild species, including wolves and foxes (Zaccaria et al., 2016; De Arcangeli et al., 2020). Circoviruses from wild animals and dogs possess up to 80% of nucleotide identity, suggesting that wild and domestic canids may share related circoviruses (De Arcangeli et al., 2020).

The CanCV has been known to cause diarrhea in dogs and showed a high detection rate in Taiwan (prevalence: 28% diarrheal dogs and 11.9% healthy dogs - p < 0.001) (Hsu et al., 2016). CanCV may also be presented as a co-infection with other viral pathogens, such as canine parvovirus, canine distemper virus and canine adenovirus, in which case it is associated with more severe gastrointestinal manifestations (Thaiwong et al., 2016; Anderson et al., 2017). Despite of the well documented presence of CanCV in gastroenteritis cases, this virus has been further detected in respiratory diseases and in some cases of vasculitis (Li et al., 2021; Decaro et al., 2014; Anderson et al., 2017).

In this study, we investigated the presence of CanCV in dogs with diarrhea in Belém, northern Brazil. Sequencing techniques and the metagenomic approach were used along with the temporal phylogenetic analysis of the CanCV to describe the genetic heterogeneity of the viral communities and the viral diversity.

## Material and methods

### Study design, animal description and ethical considerations

The present research is a cross-sectional and retrospective study performed in dogs (*Canis lupus familiaris*) from kennels of the Zoonoses Control Center (CCZ) and the Federal Rural University of Amazônia (UFRA) at the city of Belém, in the state of Pará, northern Brazil. This study involved the collection of fecal samples and rectal swabs from asymptomatic (absence of diarrhea in n = 56) as well as symptomatic (presence of diarrhea in n = 91) animals.

This study was submitted to the Ethics Committee for the Use of Animals of Instituto Evandro Chagas, certificate nº 21/2021.

### Sample processing and nucleic acid extraction

The fecal samples from the dogs were analyzed individually (fecal suspensions for the PCR detection of CanCV) and in pools (detection with the viral metagenomic approach - NGS). Suspensions were prepared with 1 g of feces for 1 mL of the 0.01M pH 7.2 Tris HCl/Ca^++^ solution, being centrifuged for 10 minutes at 4000 xg and stored at -20°C until the viral DNA was extracted.

The pooled samples consisted of the grouping of 05 to 14 biological samples, according to the following criteria: a) equimolarity - samples with equivalent or approximate nucleic acid mass (ng/µL) obtained after quantification; b) collection site - samples from the same kennels (UFRA and CCZ).

The nucleic acids were extracted with the PureLink™ Viral RNA/DNA Mini Kit (Invitrogen, California, United States) and quantified by fluorometry, using the Qubit^®^ dsDNA BR Assay Kit on the Qubit Fluorometer^®^ 2.0 equipment (Invitrogen, California, United States).

### Circovirus PCR assay validation

The CanCV detection was performed by PCR targeting the rep gene, and the assay was validated with different parameters of verification and optimization, such as: a) concentration of enzymes and cofactors; b) design, concentration, determination and amplification of artificial positive control (used in all reactions); c) verification of the primers annealing (Rev 533/For genomic) in relation to the prototype strain of canine circovirus (MT293521) with the Geneious R10 program; d) establishment of the primers annealing temperature after performing a concentration gradient PCRusing a Veriti™ 96-Well Thermal Cycler (Applied Biosystems, Massachusetts, United States).

After carrying out a concentration gradient and determination of the optimal annealing temperature, PCR assays were performed according to the concentrations and cycling conditions described in Table 1 and 2, respectively. Samples with 533 pb amplicons were considered positive for Canine Circovirus.

**Table 1:** Concentrations and volumes of reagents used in PCR reactions for CanineCV detection.

Reagents	Concentrations	Volume 1X
Ultrapure water	-	15.2 μl
DNTP*	40 mM	2.0 μl
Buffer 10X	10x	2.5 μl
MgCl_2_**	50 mM	0.8 μl
Primer Rev 533	20 pmol	0.5 μl
Primer *For Genomic*	20 pmol	0.5 μl
Taq DNA polymerase	5U/ μl	0.5 μl

* DNTP - Phosphate deoxyribonucleotides

** Mgcl2 - Magnesium chloride.

**Table 2:** PCR cycling conditions for CanineCV detection.

Parameters	Temperatures	Time
Initial denaturation	94°C	5 minutes
Amplification cycles (40x)		
Denaturation	94°C	30 seconds
Annealing	55°C	1 minute
Extension	72°C	1 minute
Final extension	72°C	5 minutes

### Sequencing

Products were purified using the PureLink™ Quick Gel Extraction Kit (Invitrogen, California, United States). The Sanger sequencing was performed with Rev 533/For genomic primers for the *rep* gene region, using the BigDye Terminator v 3.1 Cycle Sequencing kit (Applied Biosystems, Foster, USA) on the ABI 3130 DNA Sequencer platform (Applied Biosystems, Foster, USA).

### Genomic libraries and Next Generation Sequencing

Genomic libraries were made by enzymatic fragmentation from 50 ng of DNA using the Ion Xpress Plus Library kit, according to the manufacturer's recommendations. After fragmentation (Ion Shear™ Plus Enzyme Mix II), connection of adapters (P1 Adapter and barcodes), amplification (Platinum™ PCR SuperMix High Fidelity and Library Amplification Primer Mix) and several stages of purification by magnetic beads (AMpure XP beads), the 400-base-read libraries were quantified using qPCR (Ion Library TaqMan™ Quantitation Kit). The template preparation and chip loading (libraries at 100 pmol and Ion 530 Chip) was performed in Ion Chef system. Then sequencing was performed by semiconductor-based next-generation sequencing system, in Ion GeneStudio™ S5 System (Thermo Fischer, Massachusetts, United States).

### Pre-Processing, Taxonomic identification and Assembly of Genomes

The reads were trimmed using the fastp tool (Chen et al., 2018) and de novo assembled using Megahit software (Li et al., 2015) with the NR database (NCBI non-redundant protein database). Kraken 2 program was used for taxonomic classification, with interactive analysis performed in Pavian R and Krona (Ondov et al., 2011), which allow interactive visualization of data and estimation of the abundance of microorganisms. The mapping and editing of viral reads and contigs were performed using the Geneious R10 program (Kearse et al., 2012).

### Phylogenetic and Evolutionary analysis

To construct of MV and time-scaled phylogenetic trees, datasets were formed of full or near-full CanCV sequences available in GenBank. Phylogenetic inferences were performed by maximum likelihood (MV) conducted in the programs Geneious R10 program and MEGA X (Kearse et al., 2012; Kumar et al., 2018).

The evolutionary rates (nucleotide substitutions/site/year), the most recent common ancestor (TMRCA) and geographic origin were estimated using the Bayesian Markov Chain Monte Carlo approach implemented in BEAST v1.10.8 package (Drummond et al., 2012). The GTR+I+G4 substitution rate model was used (Drummond et al., 2006). To evaluate the most appropriate molecular clock, six tree prior were tested (three relaxed clock and three strict clock), and bayes factor, based in path sampling (ps) and stepping stone (ss), for indicate the best model fit. A final clock ran in triplicate with 50 million generations. The convergence of MCMC chains was checked using Tracer v.1.7.2 (Rambaut et al., 2018), ensuring the effective sample size (ESS) values greater than 200. The trees were summarized using Treannotator (burn-in 10%) and visualized into FigTree v1.4.2 (Rambaut & Drummond, 2010).

### Statistical analysis

For statistical analysis, the chi-square test was performed in the Jamovi v2.3 (The Jamovi project - 2022) program considering p<0.01 as significant.

## Results

During April 2019 to March 2020, 147 fecal samples were collected, with 56.5% (83/147) from UFRA and 43.5% (64/147) from the CCZ. Detection assays were performed after the validation of the PCR reaction by determining the enzyme concentration, Tm and annealing of oligonucleotides (55°C), with the PCR cycling conditions.

**Figure 1:**
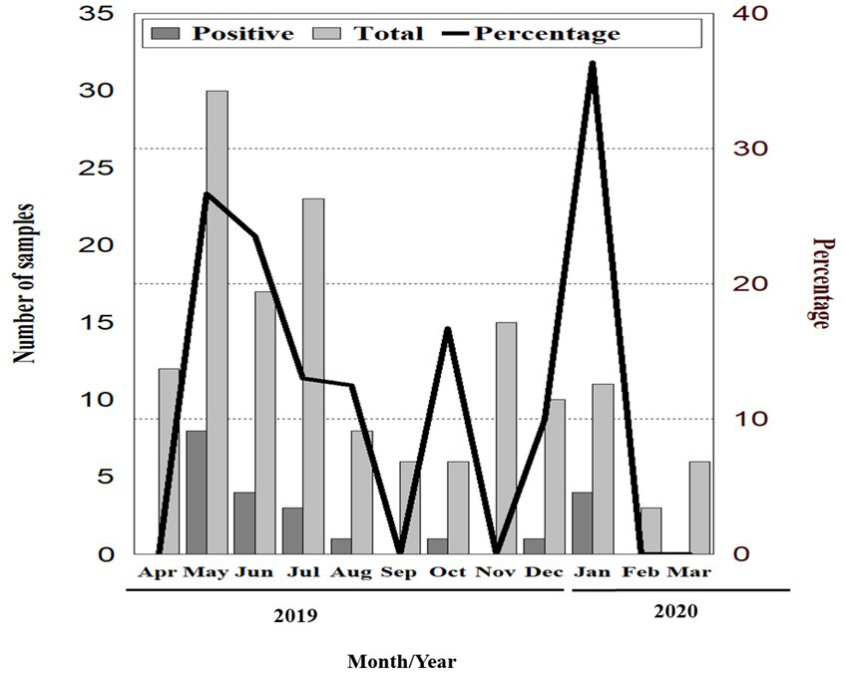
Temporal distribution of positive cases for canine circovirus in feces of dogs collected in two kennels, between April 2019 to March 2020.

All of the fecal specimens collected were tested by PCR and CanCV was detected in 15% (22/147) of the analyzed samples, with a higher incidence in diarrheal dogs under one year of age (ꭓ^2^ - p < 0.01) (Table 3). The highest monthly detection rate was verified in May and June/2019, followed by January/2020, with 26.7% (8/30), 23.5% (4/17) and 36.4% (4/11), respectively (Figure 1).

**Table 3:** CanineCV prevalence rates by age detected in feces of dogs with or without diarrheal, collected in two kennels, between April 2019 to March 2020.

Age group (years)	Diarrheal dogs Pos/total (%)	Healthy dogs Pos/total (%)	Total (%)
0-1	12/34 (35.3)	0/10	12/44 (27.3)
1-8	4/41 (9.8)	1/11(9.1)	5/52 (9.6)
> 8	0/8	0/5	0/13
NI	4/8 (50)	1/30 (3.3)	5/38 (13.2)
Total	20/91 (22)	2/56 (3.6)	22/147 (15)

**Figure 2:**
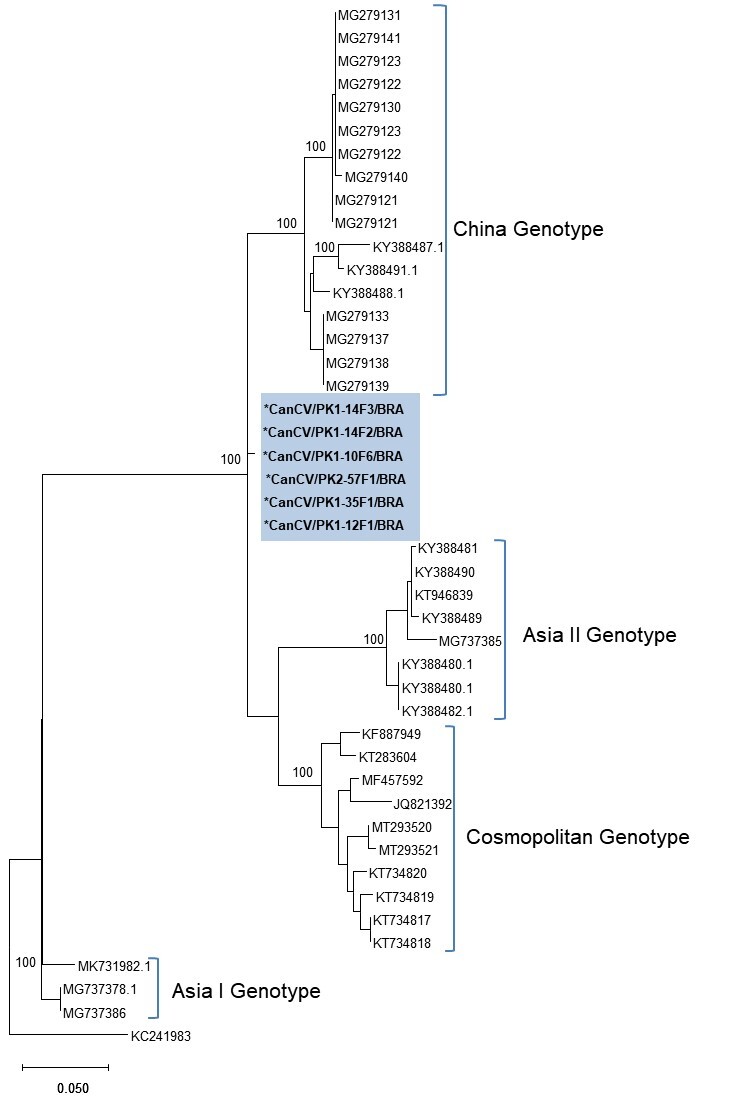
Phylogenetic evolutionary tree of canine circovirus demonstrating clades from China (including the samples from this study); Cosmopolitan; Asia I and Asia II. This analysis involved 45 nucleotide sequences. Evolutionary analyses were performed on MEGA X. Strains isolated in this study are in bold.

The maximum likelihood of the rep gene (533 nt) phylogenetic tree demonstrated that the strains detected in this study were grouped into a subclade of the Chinese genotype, distributed among the genotype strains from China and Asia II (Figure 2). The nucleotide identity was observed using a distance matrix (percentage of identical bases shown as % identity), and the samples of this study presented 99.54 - 100% of nucleotide homology. Both the samples of the present study and the genotype from China showed a nucleotide identity of 95.05 - 95.86%. The evolutionary history was inferred using the Maximum Likelihood method and the GTR model, with a discrete Gamma distribution to model the evolutionary rate differences between sites (10 categories +G, parameter = 0.3417). The nucleotide sequences obtained from our study were deposited in the GenBank with the accession numbers OP093960 - OP093965.

**Figure 3:**
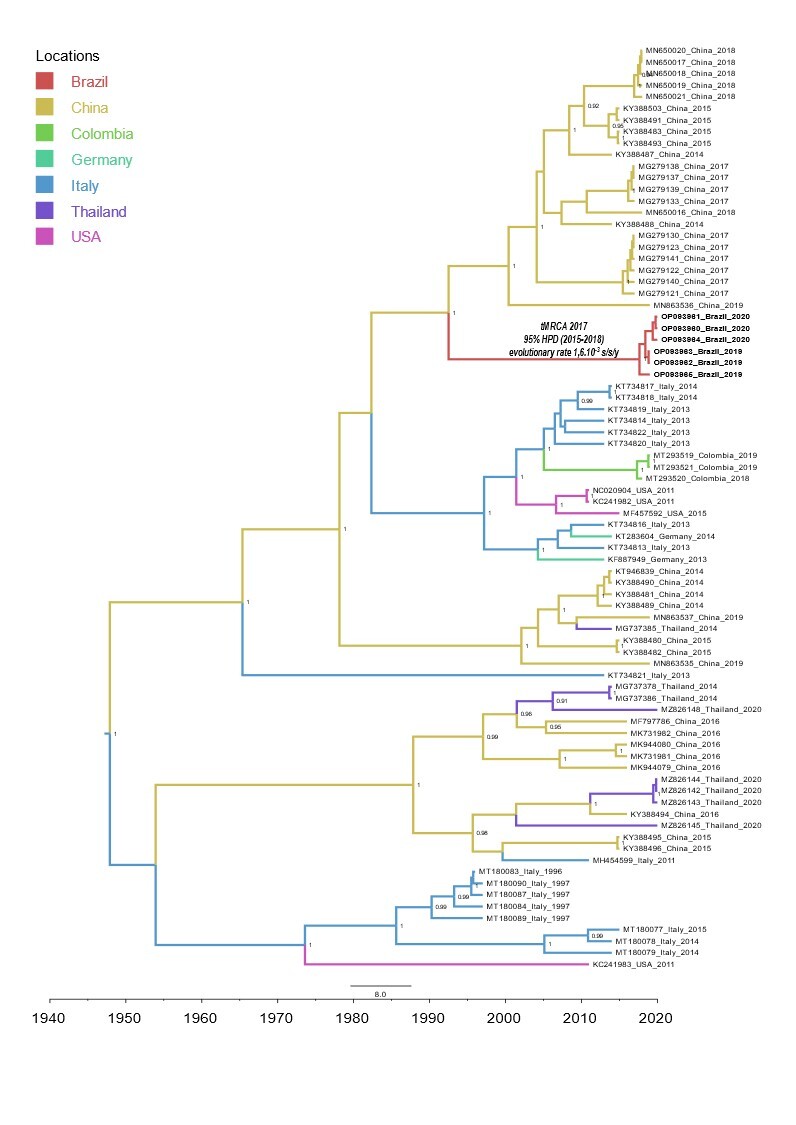
Time-scaled phylogenetic tree based on the *rep* gene of circovirus strains. The tree was constructed using the Bayesian Markov Chain Monte Carlo (MCMC) method and the GTR + I + G nucleotide substitution model. Branches are colored according to local and the distinct circovirus clades. Strains isolated in this study are in bold. The scale bar represents the unit of time (years).

Different models of molecular clock were applied to investigate the temporal evolutionary dynamics of CanCV, with the GMRF Bayesian Skyride presenting the best performance. The model selection was made by comparing the Bayes factor according to the PS/SS parameters to support decision making. Thus, through Bayesian inference (Bayes factor), the strains detected in this study showed similarities to the Chinese genotype, with the TMRCA (Time to the Most Recent Common Ancestor) estimated in 2017 (95% HPD: 2015-2018). Spatial and temporal analysis suggests that the studied strains probably were originally from China and are currently differentiating at an evolutionary rate of 1.6 x 10-3 s/s/y (Figure 3).

A total of 20-25 GB of data were obtained with the next generation sequencing of each pooled sample, an average of 0.2 to 1 million reads were generated and 196,437 contigs were obtained after assembly (viral contigs ranged from 0,02 to 1,486%). The raw data was compared with the genomic sequences available in the GenBank (6680 *Circoviridae* genomes) to identify CanCV reads by reference mapping. Circovirus positive pooled samples showed less than 5% of mapped reads and no complete genome of CanCV was obtained.

**Figure 4:**
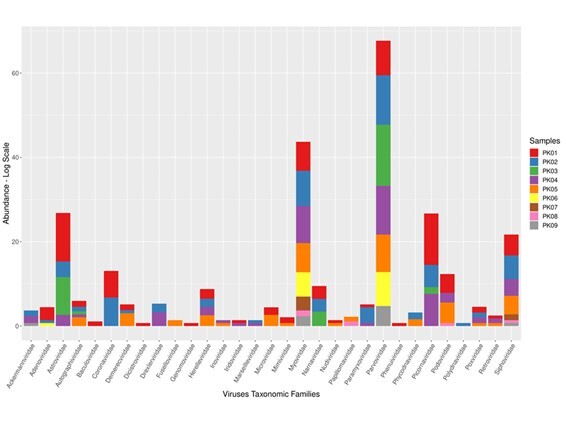
Composition of fecal virome detected in dogs from Belem, Para State, Brazil. The abundance of different virus families between nine pooled samples is demonstrated in logarithmic scale.

We assessed the percentage of sequence reads from different viral families in nine libraries and the metagenomic data was organized in a logarithmic scale to represent the abundance of viral families (Figure 4). The diversity data analysis was performed for the Family taxonomic level, with the objective of evaluating the diversity of the set of viruses observed in the different samples.

Thirty-one viral families were identified, thirteen of which from animal viruses (vertebrates and/or invertebrates), twelve bacteriophages, three insect-specific viruses, two protozoan viruses (amoeba viruses), and one fungal virus. The most abundant animal virus family was the Parvoviridae (85-99.6% of the total reads of the pooled samples PK01-06 and PK09), followed by the virus families Picornaviridae (0.14-65.5% of the total reads of the pooled samples PK01, PK02, PK04), Astroviridae (32.7% of the reads of the pooled sample PK01) and Coronaviridae (0.2-0.7% of the total reads of the pooled samples PK01 and PK02). Other families of pathogenic viruses were also present, such as the *Adenoviridae* (pooled samples PK01-2 and PK06) and the Papillomaviridae (pooled samples PK05 and PK08) (Figure 4).

The viral families including Paramyxoviridae, Poxviridae, Phenuiviridae, Retroviridae, Genomoviridae, Iridoviridae and Nudiviridae presented only a few sequences reads, with little representation in the final analysis.

## Discussion

Canine gastroenteritis is associated with several viral agents and CanCV is proven to be an emerging enteric virus in several continents (Rodrigues et al., 2018; Beikpour et al., 2022). In the present study, we investigated the CanCV circulation in the northern region of Brazil, being detected in 15% of the analyzed cases (Weber et al., 2018). In further studies carried out in southeastern Brazil the observed prevalence (1.34%) was smaller, Niu et al. (2020) detected CanCV in 15.6% of diarrheal dogs in China, and in Colombia, 16.6% of the animals with hemorrhagic diarrhea were infected by the referred virus (Dowgier et al., 2017; Giraldo-Ramirez et al., 2020).

The CanCV was more frequently detected in animals with less than one year old that presented gastrointestinal symptoms (p < 0.01). This information reinforces the hypothesis that this agent is one of the causes of gastroenteritis in dogs in this age group, according to the results obtained by Niu et al. (2020) and Beikpour et al. (2022), who also observed a positive association between the circovirus infection and diarrhea in dogs.

The strains circulating in the northern region of Brazil belong to the Chinese genotype, with nucleotide identity of 95.05 to 95.86%. In analyzes performed in other Brazilian locations, it was found that the strains circulating in the canine serum collected in Paraiba, northeast Brazil, were associated with genotypes from the United States, China and Italy. In Blumenau, south of the country, the complete genome of a Brazilian CanCV (D1056) was obtained from a dog with diarrhea and grouped with a strain from the United States (Cruz et al., 2020).

Studies conducted in Argentina and Colombia detected the circovirus cosmopolitan genotype strains with a variation of 83-98% of nucleotide identity (Kotsias et al., 2019; Giraldo-Ramirez et al., 2020). In Iran, the data from the detection and full-genome characterization of CanCV in swab samples collected from non-diarrheic dogs was described as presenting a 8.9% prevalence by real-time PCR assay, with sequences belonging to a separate clade and nucleotide identity ranging from 80.8% to 100%, in comparison to other sequences in the GenBank database (Beikpour et al., 2022).

The phylogenetic and temporal evolutionary analysis evidenced that the strains detected in this study came from China and evolved forming a distinct clade, probably due to selection and adaptation pressures (Zaccaria et al., 2016). The TRMCA was calculated computationally with MCMC-based methods, involving all human circovirus sequences available in the GenBank, and these parameters were used to estimate the ancestral virus of all the sampled cases with the same host, representing the time of the initial dispersion event which led to the spread of the circovirus human strains. The TRMCA describes the lower temporal limit of the strain circulation in the sampled population, always providing an earlier estimate than the detected circulation (Zhou & Teo, 2016).

This analysis reinforced the hypothesis of the circulation of four globally reported genotypes: three genotypes with phylogeographic patterns (China, Asia I and II genotypes), in which ancestral strains evolved independently; and other genotypes that demonstrate the transmission of CanCV between canine populations worldwide (Cosmopolitan genotype).

In addition to the temporal and evolutionary characterization of CanCV strains, we also provide information about the composition of the canine fecal virome using a shotgun metagenomic approach to identify the circulation of other viruses, describing the viral communities in the fecal samples of dogs with diarrhea by next generation sequencing analysis. Thus, it is possible to visualize the presence of several viral families after the taxonomic identification of reads, including the Parvoviridae, the Picornaviridae, the Astroviridae and the Coronaviridae, which were the most abundant.

The canine fecal virome is still poorly studied, therefore, we have little information on the composition of the fecal virome of dogs with diarrhea. The viral metagenomic approach was used to investigate the fecal virome in Chinese raccoon dogs and many DNA or RNA virus families were observed, including the *Circoviridae*, the *Smacoviridae*, the *Genomoviridae*, the *Parvoviridae*, the *Picornaviridae*, the *Astroviridae* and the *Hepeviridae* (Yang et al., 2021). Fecal virome of healthy dogs and dogs with acute diarrhea were studied in Australia, detecting various viral families: *Astroviridae* (more abundant), *Picornaviridae* and *Caliciviridae* in dogs with acute diarrhea; *Coronaviridae*, *Reoviridae*, *Parvoviridae*, *Adenoviridae* and *Papillomaviridae* in healthy dogs (Moreno et al., 2017).

The fecal virome of asymptomatic red foxes from peri-urban areas in central Croatia was evaluated with the detection of the picobirnavirus, the parvovirus and a new fox circovirus highly similar to the dog circoviruses identified in diseased dogs in USA, Italy and United Kingdom (Lojkić et al., 2016).

Studies involving other methodological approaches also demonstrate that the CanCV is often detected in association with other enteric viruses, such as the CAdV, the CPV and the CDV (Thaiwong et al., 2016; Mortari et al., 2022). In Italy there were reports of co-infection cases with CPV, CAdV and CanCV in fecal specimens from dogs with enteritis (Balboni et al., 2022). In Thailand, the prevalence was 19.8% positive cases for co-infection of CPV and CanCV, with a death rate of 50% (Tuong et al., 2021). These high prevalence data of co-infections may be due to the CanCV blocking of the immune response, promoting viral replication and increasing the severity of clinical symptoms (Hao et al., 2022).

In the present study we report the molecular epidemiology of the CanCV and fecal virome in dogs living at public shelters in the northern region of Brazil. Our results confirm the circulation of several viruses families that cause gastrointestinal diseases in dogs, highlighting the CanCV as an emerging and epidemic virus.

## Conclusion

This comprehensive study is the first research to describe the detection and the molecular characterization of CanCV in dogs of the Amazon region, providing new insights into viral detection, viral diversity and fecal virome in dogs. The results presented herein demonstrate a high prevalence of CanCV as a source of diarrhea in dogs. Additionally, our results reinforce the information regarding the circulation of four CanCV genotypes, which have evolved and been distributed worldwide, probably due to local selection and adaptation pressures. These findings provide useful information about enteric infections in dogs, contributing to the surveillance and monitoring of the referred virus, since its circulation adds risks to the canine population.
